# 

**Published:** 1987-05

**Authors:** 


NO.2 1987

1--------SEQ: B33740000

BRISTOL MEDICO-CHIRURGICAL

JOURNAL

### BRISTOL MEDICO-CHIRURGICAL JOURNAL

VOLUME 102(ii) MAY 1987

NATIONAL LIBRARY
AUG 11 1987
OF MEDICINE

Abdominal symptoms in general practice. p 33
Academic practice or primitive physic. p 42
Epanutin and Hodgkin's disease. p 35
The dangers of tapioca consumption. p 37
Haematemesis in gastric carcinoid. p 40
Oesophageal pain mimicking cardiac ischaemia. p 41

Putting the boot in. p 44
On Gardening. p 56
Etc. etc.

IN ASSOCIATION WITH
BRISTOL MEDICINE
THE MEDICAL JOURNAL OF THE SOUTH WEST

